# Neural crest and the origin of species‐specific pattern

**DOI:** 10.1002/dvg.23219

**Published:** 2018-08-08

**Authors:** Richard A. Schneider

**Affiliations:** ^1^ Department of Orthopedic Surgery University of California at San Francisco, 513 Parnassus Avenue S‐1161 San Francisco, California

**Keywords:** cranial neural crest, craniofacial development, evolutionary‐developmental biology, quail‐duck chimeras, quck, species‐specific pattern, tissue‐interactions

## Abstract

For well over half of the 150 years since the discovery of the neural crest, the special ability of these cells to function as a source of species‐specific pattern has been clearly recognized. Initially, this observation arose in association with chimeric transplant experiments among differentially pigmented amphibians, where the neural crest origin for melanocytes had been duly noted. Shortly thereafter, the role of cranial neural crest cells in transmitting species‐specific information on size and shape to the pharyngeal arch skeleton as well as in regulating the timing of its differentiation became readily apparent. Since then, what has emerged is a deeper understanding of how the neural crest accomplishes such a presumably difficult mission, and this includes a more complete picture of the molecular and cellular programs whereby neural crest shapes the face of each species. This review covers studies on a broad range of vertebrates and describes neural‐crest‐mediated mechanisms that endow the craniofacial complex with species‐specific pattern. A major focus is on experiments in quail and duck embryos that reveal a hierarchy of cell‐autonomous and non‐autonomous signaling interactions through which neural crest generates species‐specific pattern in the craniofacial integument, skeleton, and musculature. By controlling size and shape throughout the development of these systems, the neural crest underlies the structural and functional integration of the craniofacial complex during evolution.

## 1 INTRODUCTION

The notion that neural crest cells generate species‐specific pattern has a long and colorful history. Some of the earliest indications first arose from surgical transplantation experiments designed to exploit pigment variations in amphibian embryos. Around the beginning of the 20th Century, embryologists such as Born (1896), Harrison (1898, 1903), and Spemann (1918) pioneered the use of chimeras, that is combining embryonic components from distinct animal species, to follow the movements and fates of cells, and understand the inductive properties of tissues (Harrison, 1935; Mangold, 1923; Mangold & Seidel, 1927; Noden, 1984; Spemann, 1938; Spemann & Mangold, 1924). Their reliance on intrinsic differences in the number, distribution, and color of intracellular pigment granules as a means to keep track of donor versus host tissues was actually a proxy for a neural crest‐derived lineage (i.e., melanocytes), something which was suggested by Harrison (1910) and others but which remained debatable at the time (Dorris, 1938; DuShane, 1934, 1935, 1938, 1939; Harrison, 1969; Holtfreter, 1933; Raven, 1931). Soon thereafter, numerous efforts were underway to determine the extent to which neural crest cells establish inter‐ and intra‐specific pigment patterns and to sort out the effects and/or role of interactions with epidermis (Clark Dalton, 1950; Harrison, 1935; Hörstadius, 1950; Macmillan, 1976). For example, neural crest transplants among the tiger salamander, spotted salamander, or white and black strains of the Mexican salamander revealed that the “characteristic adult spots of the graft are in most cases distinctly different from those of the host, and are similar to those of donor adults” (DuShane, 1935, p. 25). Other interspecific transplants also confirmed this finding (Twitty, 1936, 1945; Twitty & Bodenstein, 1939). Thus, what became evident was that “the type of pattern as a whole depends upon qualities intrinsic to the crest‐cells” (Hörstadius, 1950, p. 75). What remained unknown was if the neural crest was playing a comparable role in providing species‐specific patterning information for any of its other derivatives, something that chimeras could help resolve.

Chimerism was all the rage at the dawn of experimental embryology given its great potential to reveal morphogenetic mechanisms during normal development that lead to the progressive integration of ectoderm, mesoderm, and endoderm; but also, because chimerism could provide a window into the developmental basis for evolutionary variation among species. In his comprehensive review, “Heteroplastic Grafting in Embryology” Harrison (1935) claimed with regard to making chimeras that, “the applicability of the method rests upon the fact that there are related species that differ from one another in pigmentation, rate of growth and development, ultimate size, relative time of appearance of organs, or even in the presence or absence of organs, while at the same time their tissues show a mutual tolerance when combined in one organism” (Harrison, 1969, p. 216). Results of these chimeric transplants among different taxa (i.e., species, genera, families, and even orders such as frogs and salamanders) indicated that some donor tissues did not convey species‐specific information.

Harrison (1935) further described conclusions from a broad range of studies and stated that, “In general, inducers are not specific.…But the induced organ has entirely the character of the species from which it is developed” (Harrison, 1969, p. 217). For example, in classic “organizer” studies (Spemann, 1918, 1938; Spemann & Schotte, 1932), where some of the donor tissues were from salamander endoderm, the induced mouth parts remained frog‐like leading to Spemann's purported description of the conversation between the host ectoderm tissue and its endoderm inducer, “you tell me to make a mouth; all right, I'll do so, but I can't make your kind of mouth; I can make my own and I'll do that” (Harrison, 1933, p. 318).

In contrast, when entire limb buds were exchanged between two species of salamanders with very different rates of development or where the limbs themselves varied greatly in size, the resultant chimeras always had limbs like that of the donor in terms of timing of maturation and morphology (Detwiler, 1930; Harrison, 1915, 1917, 1924; Schwind, 1932; Swett, 1930). Grafts of other embryonic rudiments and whole organs such as the eye, ear, heart, teeth, or gills produced equivalent results (Copenhaver, 1930; Harrison, 1929, 1935; Huxley, 1932; Kaan, 1930; Richardson, 1932; Stone, 1930; Twitty, 1930, 1932, 1934; Twitty & Schwind, 1931), with the conclusion being that somewhere within a composite organ resided the source of species‐specific pattern.

Subsequent explorations of the relative contributions of the constituent parts of these composite organs began to shed light on this issue, particularly with regard to the requisite role for derivatives of each of the three germ layers. In the case of the eye, the lens (from surface ectoderm) and the optic cup (from neural ectoderm) were mutually regulating (Harrison, 1929, 1935; Stone & Dinnean, 1940; Twitty, 1930, 1932), whereas host eye muscles (from paraxial mesoderm) were subservient to the eye itself and would accommodate the size, orientation, and location of the donor eye (Twitty, 1930, 1932, 1934, 1966). In the case of the forelimb, the mesoderm, which produces the muscles and skeleton, would always determine its size, shape, and growth rate, whereas the ectoderm had limited influence (Harrison, 1935; Rotmann, 1931, 1933; Schwind, 1931, 1932).

Like what had been observed for pigment patterns, the gills or branchial system of the pharynx provided clear evidence for a dominant role of neural crest‐derived mesenchyme not only in generating the jaw and gill cartilages themselves (Landacre, 1921; Platt, 1891, 1893, 1898; Stone, 1922, 1926, 1929) but also in determining their species‐specific pattern (De Beer, 1947; Harrison, 1935). In addition to the neural crest skeletal derivatives, the gill arches contain a pouch lined by endoderm, muscles from the mesoderm, and an outer epithelium from the ectoderm (De Beer, 1937; Goodrich, 1913, 1918, 1930). Numerous grafting experiments of each of these constituents either separately or in various combinations (Adams, 1931; Harrison, 1921, 1935; Holtfreter, 1936; Rotmann, 1931, 1933; Severinghaus, 1930; Spemann, 1921; Stone, 1932) showed that the ectoderm, mesoderm, and endoderm had mixed and inconsistent effects on the size, shape, and rate of development of the external gills.

In stark contrast, were the neural crest cells, which according to Harrison (1935) were “Far more conclusive” and had “a profound effect upon the development of the branchial system, particularly the visceral skeleton” (Harrison, 1969, p. 233). For example, transplants of neural crest from a larger species of salamander in place of neural crest from a smaller species gave rise to pharyngeal arches with the size and shape of the donor species (De Beer, 1947; Harrison, 1933; Hörstadius, 1950). Other neural crest transplants among frogs and salamanders also produced donor‐like cartilages in the pharyngeal arches and jaw skeleton (Andres, 1949; Fassler, 1996; Hall & Hörstadius, 1988; Hörstadius & Sellman, 1941, 1946; Noden & Schneider, 2006; Raven, 1931, 1933, 1935; Spemann & Schotte, 1932; Wagner, 1959). Early on, such work suggested to scientists like Raven (1933), as conveyed by Hörstadius (1950) that, “the neural crest in the head might have a special task in connection with its movements, as a carrier of inductive influences” (p. 93). This classic body of literature demonstrated clearly that, “transplanted neural crest cells express a species‐specific patterning that is an intrinsic property of the skeletogenic cells” (Hall, 1999, p. 71). Moreover, this initial work garnered a much deeper appreciation for the hierarchical levels of organization within these complex developmental organ systems and pointed to the key role for neural crest cells in the evolution of species‐specific morphology.

By the turn of the 21th Century, other transplant experiments in non‐amphibian taxa such as among mouse, human, chick, or quail (Cohen et al., 2016; Fontaine‐Perus, 2000; Fontaine‐Perus & Cheraud, 2005; Fontaine‐Perus, Cheraud, & Halgand, 1996; Fontaine‐Perus et al., 1997; Kirby, Stadt, Kumiski, & Herlea, 2000; Lwigale & Schneider, 2008; Mitsiadis, Caton, & Cobourne, 2006; Mitsiadis, Cheraud, Sharpe, & Fontaine‐Perus, 2003; Pudliszewski & Pardanaud, 2005; Serbedzija & McMahon, 1997); among divergent species of birds including quail, chick, duck, and emu (Ealba et al., 2015; Eames & Schneider, 2005, 2008; Fish & Schneider, 2014a, 2014b; Fish, Sklar, Woronowicz, & Schneider, 2014; Hall et al., 2014; Jheon & Schneider, 2009; Le Douarin, Dieterlen‐Lievre, Teillet, & Ziller, 2000; Merrill, Eames, Weston, Heath, & Schneider, 2008; Schneider, 2005, 2015; Schneider & Helms, 2003; Sohal, 1976; Solem, Eames, Tokita, & Schneider, 2011; Tokita & Schneider, 2009; Tucker & Lumsden, 2004; Woronowicz, Gline, Herfat, Fields, & Schneider, 2018; Yamashita & Sohal, 1986); as well as between Mexican cavefish and surface fish (Yoshizawa, Hixon, & Jeffery, 2018), reinforced the conclusion that species‐specific pattern in the craniofacial complex is largely driven by the neural crest. Harrison (1969) argued that such an ability is due to “congenital specific factors” that “control the relative growth rate” (p. 31) of grafts. Noden (1984) correspondingly observed that, “while quail tissues differentiate more rapidly, they generally form smaller skeletal structures than do chick tissues” (p. 274), which in the case of quail‐chick chimeras leads to the formation of shorter quail‐like upper and lower portions of the beak, depending on from where along the neural tube the quail donor neural crest cells are derived. In other words, donor neural crest cells keep track of their intrinsic rates of development, and that appears to have direct implications for the generation of species‐specific size and shape in the craniofacial complex.

**Figure 1:**
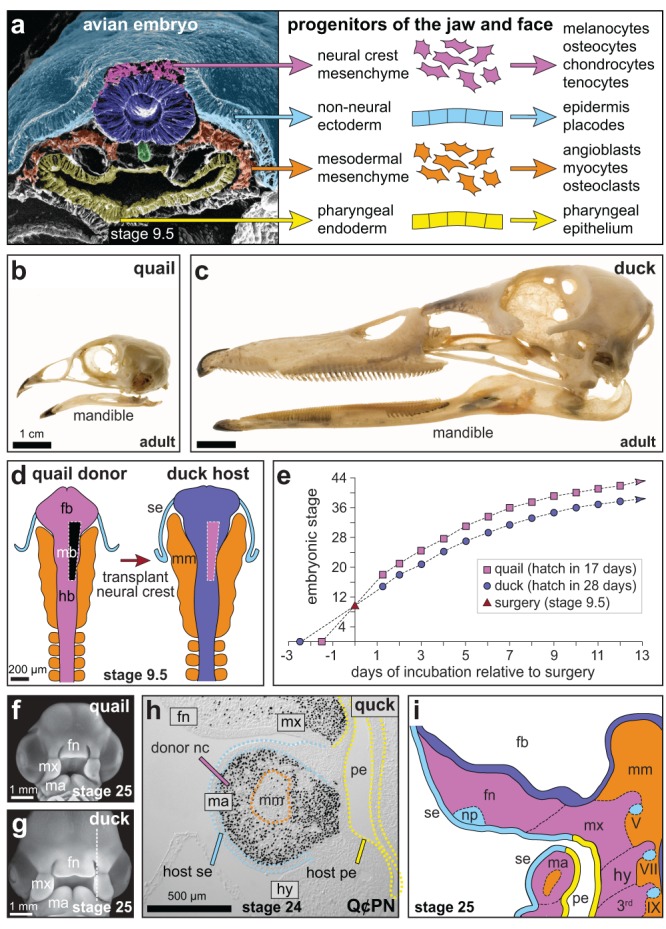
**(a)** Pseudocolored scanning electron micrograph of a chick in cross‐section [modified from Tosney (1982)] through the midbrain/hindbrain boundary and first pharyngeal arch showing progenitors of tissues in the jaw and face. Modified from Ealba et al. (2015). **(b)** Skull and lower jaw (mandible) of an adult quail and **(c)** duck in lateral view. Modified from Tokita and Schneider (2009). **(d)** Unilateral transplant of presumptive quail neural crest from the posterior forebrain (fb), midbrain (mb), and anterior hindbrain (hb) into a duck host. Non‐neural surface ectoderm (se) and mesodermal mesenchyme (mm) are shown in dorsal view. Modified from Eames and Schneider (2008). **(e)** Distinct maturation rates of quail (pink squares) versus duck (purple circles) after being stage‐matched at HH9.5 for surgery (red triangle on the Y‐axis) result in quail donor cells remaining accelerated by approximately three stages within 2 days after surgery relative to the duck host [modified from Eames & Schneider (2005)]. **(f)** By stage 25, the frontonasal (fn), maxillary (mx), and mandibular (ma) primordia of quail and **(g)** duck appear similar in shape but not in size (frontal view). Modified from Schneider (2005). **(h)** Sagittal section (in plane of white dashed line in panel g) in a chimeric quck through the maxillary (mx) and mandibular (ma) region showing quail donor cells labeled with Q¢PN (black nuclei). Duck‐host surface ectoderm (se), pharyngeal endoderm (pe), mesodermal mesenchyme (mm) are unlabeled. The hyoid arch (hy) is also negative since its precursors were not transplanted. Modified from Ealba and Schneider (2013). **(i)** By HH25, the frontonasal (fn), maxillary (mx), mandibular (ma), and hyoid (hy) primordia (sagittal view) are surrounded by surface ectoderm (se), pharyngeal endoderm (pe) and forebrain neuroepithelium (fb), and contain contributions from the neural crest, nasal placode (np), and cranial ganglia (V, VII, IX). Mesodermal mesenchyme (mm) produces muscles, vascular endothelium, and some skeletal tissues. Modified from Schneider (2005)

Discerning exactly how neural crest accomplishes such a seemingly complicated task and pinpointing precise morphogenetic mechanisms that ultimately function as determinants of species‐specific pattern, has been a goal of work from our lab over the past 15 years. Somewhat systematically, we have been investigating the extent to which the cranial neural crest directs the patterning of its own derivatives (e.g., cartilages, bones, tendons), as well as those arising from ectoderm (e.g., feathers and egg teeth) and mesoderm (e.g., muscles, blood vessels, osteoclasts) in order to understand how the major systems of the craniofacial complex become structurally and functionally integrated during development and how they become modified during evolution (Figure 1a). Such work, which has helped illuminate the origin of species‐specific pattern, is summarized and contextualized in the sections below.

## 2 ORIGIN OF SPECIES‐SPECIFIC VERSUS SPECIES‐GENERIC ASPECTS OF PATTERN

When describing species‐specific pattern, what is typically meant are those relatively unique morphological or behavioral features of an organism that often appear well‐suited to meet certain functional, ecological, sexual, or other kinds of selective pressures. Moreover, such features can be defining and used to distinguish one species (or higher taxonomic level) from another. In this context, and in terms of morphology, a type of species‐specific pattern that has long been of central concern pertains to changes in size and shape during development and evolution (Fish & Schneider, 2014b; Schneider, 2015, 2018). This focus was most significantly catalogued and detailed over 100 years ago by Thompson (1917) in his celebrated tome, *On Growth and Form.* Using a geometric system of Cartesian coordinates, Thompson strove to describe transformations in the size and shape of organs and organisms during the growth of individuals and across different species. In so doing, he helped spawn an entire discipline of morphometrics that continues to this day (Arthur, 2006; Benson, Chapman, & Siegel, 1982; Bookstein, 1978, 1990; Gayon, 2000; Hallgrimsson et al., 2015; Marcus, 1996; Schneider, 2018; Siegel & Benson, 1982; Stern & Emlen, 1999; Zelditch, 2004).

Since Thompson, many other scientists have endeavored to address the origins of species‐specific size and shape through mathematical, theoretical, and experimental means, ultimately in search of underlying genetic, molecular, cellular, or other developmental mechanisms including allometry and heterochrony (Alberch, 1982a, 1985, 1989; Alberch, Gould, Oster, & Wake, 1979; Anderson & Busch, 1941; Atchley, Rutledge, & Cowley, 1981; Bertalanffy & Pirozynski, 1952; Clark & Medawar, 1945; Coppinger & Coppinger, 1982; Coppinger & Schneider, 1995; De Beer, 1930; De Renzi, 2009; Drake, 2011; Godfrey & Sutherland, 1995; Gould, 1966, 1971, 1977; Hersh, 1934; Huxley, 1932, 1950; Huxley & Teissier, 1936; Kermack & Haldane, 1950; Klingenberg, 1998; Lande, 1979; Lord, Schneider, & Coppinger, 2016; Lumer, 1940; Minot, 1908; Needham & Lerner, 1940; Oster & Alberch, 1982; Oster, Shubin, Murray, & Alberch, 1988; Reeve, 1950; Rensch, 1948; Roth & Mercer, 2000; Shea, 1985; Smith et al., 2015; Smith, 2003; Stern & Emlen, 1999; Von Bonin, 1937; Waddington, 1950, 1957). A common theme for much of the research on size and shape relates to those changes that occur with respect to developmental time either as a function of age or growth. Minot (1908) laid the groundwork for this perspective by emphasizing the importance of cell number, differentiation, and rates of growth in the regulation of the size of animals and/or their organs. Thompson (1952) later elaborated on this idea when stating that, “the *form* of an organism is determined by its rate of *growth* in various directions; hence rate of growth deserves to be studied as a necessary preliminary to the theoretical study of form, and organic form itself is found, mathematically speaking, to be a *function of time*” (p. 79). Thus, given that the neural crest generates species‐specific pattern in the craniofacial complex, and this pattern can be defined primarily as the size and shape of structures, then a critical insight could be gained by understanding the extent to which the neural crest controls the timing of events during development. A further question also remains, which is from where do other aspects of craniofacial pattern (i.e., those that are not necessarily species‐specific) arise?

In addition to their species‐specific pattern, structures likewise possess many more “species‐generic” aspects of pattern. These include their axial orientation (e.g., dorsal‐ventral, medial‐lateral, proximal‐distal, oral‐aboral), anatomical identity (e.g., upper versus lower jaw, eye versus ear), and tissue type (e.g., cartilage, bone, muscle, tendon, nerve). For the most part, epithelia in the craniofacial complex appear to supply the cues required for the establishment of generic pattern and express the factors necessary to maintain outgrowth of individual components. For example, signaling by ectodermal epithelium around the frontonasal process (i.e., the primordium that gives rise to the mid‐ and upper‐face) is essential for proper expansion and orientation of skeletal elements along the dorsoventral, mediolateral, and proximodistal axes (Foppiano, Hu, & Marcucio, 2007; Hu & Marcucio, 2009b; Hu, Marcucio, & Helms, 2003). Experimentally rotating epithelium in the frontonasal process can lead to mirror image duplications of upper beak structures along the dorsal–ventral axis (Helms & Schneider, 2003; Hu et al., 2003; Marcucio, Cordero, Hu, & Helms, 2005).

Similarly, endodermal epithelium that lines the pharynx is needed for the proper axial orientation, anatomical identity, and growth of cartilage and bone in the lower jaw and hyoid skeleton (Brito, Teillet, & Le Douarin, 2006; Couly, Creuzet, Bennaceur, Vincent, & Le Douarin, 2002; Crump, Maves, Lawson, Weinstein, & Kimmel, 2004; David, Saint‐Etienne, Tsang, Schilling, & Rosa, 2002; Delloye‐Bourgeois, Rama, Brito, Le Douarin, & Mehlen, 2014; Graham, 2003; Haworth et al., 2007; Kikuchi et al., 2001; Kimmel et al., 1998; Miller, Schilling, Lee, Parker, & Kimmel, 2000; Piotrowski & Nusslein‐Volhard, 2000; Ruhin et al., 2003; Veitch, Begbie, Schilling, Smith, & Graham, 1999). When endodermal epithelium is rotated surgically or removed, the associated neural crest‐derived skeleton follows accordingly (Couly et al., 2002; Haworth et al., 2007). Therefore, both ectodermal and endodermal epithelia function as local sources of signals for generic pattern that elicit and/or maintain programmatic responses from the adjacent neural crest‐derived mesenchyme (Creuzet, Couly, & Le Douarin, 2005; Ferguson, Tucker, & Sharpe, 2000; Higashihori, Buchtova, & Richman, 2010; Langille & Hall, 1993; Le Douarin, Creuzet, Couly, & Dupin, 2004; Mitsiadis et al., 2003; Richman & Tickle, 1989; Santagati & Rijli, 2003; Tak, Park, Piao, & Lee, 2017; Tucker, Yamada, Grigoriou, Pachnis, & Sharpe, 1999; Wilson & Tucker, 2004). As will be discussed later, such programmatic responses are in fact species‐specific, and they also reciprocally influence the temporal and spatial domains of expression in adjacent epithelia (Eames & Schneider, 2005; Schneider & Helms, 2003).

Pharyngeal endoderm, and neural and non‐neural ectoderm function as key epithelial signaling centers by releasing complex combinations of secreted molecules from well‐characterized pathways including Bone Morphogenetic Protein (BMP), Sonic Hedgehog (SHH), Fibroblast Growth Factor (FGF), and Wingless‐Related (WNT) that are indispensable to the proper patterning and differentiation of neural crest mesenchyme (Alvarado‐Mallart, 2005; Anderson, Lawrence, Stottmann, Bachiller, & Klingensmith, 2002; Barlow & Francis‐West, 1997; Benouaiche, Gitton, Vincent, Couly, & Levi, 2008; Cela et al., 2016; Crump et al., 2004; Ekker et al., 1995; Francis‐West, Ladher, Barlow, & Graveson, 1998; Gitton et al., 2010; Graham, 2003; Marcucio et al., 2005; Marcucio, Young, Hu, & Hallgrimsson, 2011; Pera, Stein, & Kessel, 1999; Piotrowski & Nusslein‐Volhard, 2000; Sasai & De Robertis, 1997; Schneider, Hu, Rubenstein, Maden, & Helms, 2001; Shimamura, Hartigan, Martinez, Puelles, & Rubenstein, 1995; Veitch et al., 1999; Wilson & Tucker, 2004; Withington, Beddington, & Cooke, 2001; Xu et al., 2015). Various members and targets of these pathways also become differentially regulated not only as a mechanism to support the outgrowth of the jaw and facial skeletons (Abzhanov & Tabin, 2004; Ashique, Fu, & Richman, 2002a; Chong et al., 2012; Cordero, Schneider, & Helms, 2002; Couly et al., 2002; Doufexi & Mina, 2008; Geetha‐Loganathan, Nimmagadda, Fu, & Richman, 2014; Havens et al., 2008; Helms & Schneider, 2003; Hu, Colnot, & Marcucio, 2008; Hu et al., 2003; Liu et al., 2005; MacDonald, Abbott, & Richman, 2004; Melnick, Witcher, Bringas, Carlsson, & Jaskoll, 2005; Miller et al., 2000; Mina, Wang, Ivanisevic, Upholt, & Rodgers, 2002; Nimmagadda et al., 2015; Richman, Herbert, Matovinovic, & Walin, 1997; Rowe, Richman, & Brickell, 1992; Schneider, Hu, & Helms, 1999; Schneider et al., 2001; Szabo‐Rogers, Geetha‐Loganathan, Nimmagadda, Fu, & Richman, 2008; Wada et al., 2005; Young, Chong, Hu, Hallgrimsson, & Marcucio, 2010) but also as a component of regulating species‐specific size and shape (Abramyan, Leung, & Richman, 2014; Abzhanov et al., 2006; Abzhanov, Protas, Grant, Grant, & Tabin, 2004; Bhullar et al., 2015; Brugmann et al., 2007; Brugmann et al., 2010; Cheng et al., 2017; Foppiano et al., 2007; Grant, Grant, & Abzhanov, 2006; Hu & Marcucio, 2009b, 2012; Hu, Young, Li, et al., 2015; Hu, Young, Xu, et al., 2015; Wu, Jiang, Shen, Widelitz, & Chuong, 2006; Wu, Jiang, Suksaweang, Widelitz, & Chuong, 2004; Young et al., 2014).

Other epithelial tissues that are derived from surface (i.e., non‐neural) ectoderm also function as critical patterning centers in conjunction with cranial neural crest. In particular, cranial placodes that contribute to sensory ganglia and sense organs such as the olfactory, optic, otic, trigeminal, and epibranchial systems require repeated and reciprocal interactions with adjacent neural crest mesenchyme for their proper morphogenesis (Baker & Bronner‐Fraser, 2001; Bancroft & Bellairs, 1977; Couly & Le Douarin, 1988; Francis‐West, Ladher, & Schoenwolf, 2002; Ladher, O'Neill, & Begbie, 2010; Lwigale, 2001; Pispa & Thesleff, 2003; Song, Hui, Fu, & Richman, 2004; Szabo‐Rogers et al., 2008; Webb & Noden, 1993).

Members and targets of the FGF, BMP, WNT, and other pathways mediate complex signaling interactions among the developing placodes, mesoderm, endoderm, and the neural crest, which in turn lead to the differential activation of placode‐specific sets of transcription factors (Anwar, Tambalo, Ranganathan, Grocott, & Streit, 2017; Baker, Stark, Marcelle, & Bronner‐Fraser, 1999; Brunskill et al., 2014; Depew et al., 1999; Grocott, Johnson, Bailey, & Streit, 2011; Groves & Bronner‐Fraser, 2000; Hintze et al., 2017; Jourdeuil & Taneyhill, 2018; Ladher, 2017; Ladher, Wright, Moon, Mansour, & Schoenwolf, 2005; McLarren, Litsiou, & Streit, 2003; Moody & LaMantia, 2015; Saint‐Jeannet & Moody, 2014; Steventon, Mayor, & Streit, 2014; Yang et al., 2013). In almost all of these cases, the neural crest plays an obligatory role during proper patterning and differentiation.

As neural crest cells migrate throughout the craniofacial complex and settle adjacent to these different types of epithelia they respond by expressing a broad range of transcription factors and other genes that affect their anatomical identity (Balling, Mutter, Gruss, & Kessel, 1989; Clouthier et al., 2000; Creuzet, Couly, Vincent, & Le Douarin, 2002; Depew, Lufkin, & Rubenstein, 2002; Gendron‐Maguire, Mallo, Zhang, & Gridley, 1993; Grammatopoulos, Bell, Toole, Lumsden, & Tucker, 2000; Hunt, Clarke, Buxton, Ferretti, & Thorogood, 1998; Kimmel et al., 2005; Lufkin et al., 1992; Pasqualetti, Ori, Nardi, & Rijli, 2000; Qiu et al., 1997; Rijli et al., 1993; Ruest, Xiang, Lim, Levi, & Clouthier, 2004; Schilling, 1997; Smith & Schneider, 1998; Tavares, Cox, Maxson, Ford, & Clouthier, 2017). Modulating the levels of various molecules expressed by these epithelia, such as retinoic acid and the BMP antagonist Noggin, can for example, transform one facial primordium into another (Lee, Fu, Hui, & Richman, 2001; Richman & Lee, 2003) ostensibly by altering the gene regulatory networks within the responding neural crest cells.

Moreover, combinatorial expression of homeobox genes such as those in the *Hox* cluster and other transcription factors affect the ability of neural crest cells from the posterior hindbrain to form appropriate anatomical pattern in the hyoid and subsequent arches (Couly & Le Douarin, 1990; Trainor & Krumlauf, 2000; Trainor & Krumlauf, 2001). In contrast, neural crest cells from the midbrain and anterior hindbrain that migrate into the frontonasal, maxillary, and mandibular primordia do not rely on *Hox* genes (Couly et al., 2002; Couly, Grapin‐Botton, Coltey, Ruhin, & Le Douarin, 1998; Hunt & Krumlauf, 1991; Hunt, Wilkinson, & Krumlauf, 1991). If these midbrain and anterior hindbrain populations of neural crest cells are surgically rotated by 180° in order to transpose frontonasal and mandibular precursors, they generate facial and jaw skeletons that are appropriate for their new location, which reinforces the idea that anatomical identity is established locally (Noden, 1983) in response to epithelial signals.

Along similar lines, if the *Hox* code is deleted from neural crest cells destined to form the hyoid arch either by grafting non‐*Hox*‐expressing mandibular or frontonasal neural crest in place of hyoid arch neural crest cells, or by knocking down *Hoxa2*, then hyoid arch skeletal elements become replaced by mandibular structures (Gendron‐Maguire et al., 1993; Noden, 1983; Rijli et al., 1993; Trainor, Ariza‐McNaughton, & Krumlauf, 2002; Trainor, Melton, & Manzanares, 2003). Conversely, over‐expressing *Hoxa2* in mandibular arch neural crest cells gives rise to hyoid skeletal structures instead of mandibular ones (Grammatopoulos et al., 2000; Pasqualetti et al., 2000). Also illustrating the necessity of signaling interactions between the neural ectoderm and the adjacent neural crest, *Hoxa2* is downregulated by FGF8, and ectopic expression of *Fgf8* in the hindbrain disrupts the pattern of hyoid arch structures (Creuzet et al., 2002; Trainor, Ariza‐McNaughton, et al., 2002). Thus, ongoing and reciprocal interactions between epithelia derived from the ectoderm and endoderm, and neural crest mesenchyme lead to the activation of intrinsic transcription factor modules that establish a more species‐generic type of pattern, specifically the axial orientation and anatomical identity of craniofacial structures.

Such a conclusion is further supported by experiments that alter combinatorial codes of transcription factors including the *Dlx* genes, and that genetically manipulate signaling pathways such as *endothelin*, which affect the axial pattern, outgrowth, and in some instances switch the anatomical identify of the maxillary and the mandibular primordia (Depew et al., 2002; Kuraku, Takio, Sugahara, Takechi, & Kuratani, 2010; Miller, Yelon, Stainier, & Kimmel, 2003; Sato et al., 2008; Tavares et al., 2012; Tavares et al., 2017). Notably, these molecular mechanisms and gene regulatory networks that pattern the axes and impart anatomical identity within the pharyngeal arches and other regions of the craniofacial complex have remained highly conserved across vertebrates (Cerny, Lwigale, et al., 2004; Cerny, Meulemans, et al., 2004; Depew & Compagnucci, 2008; Kuraku et al., 2010; Kuratani, 2004, 2005a, 2005b, 2012; Kuratani, Adachi, Wada, Oisi, & Sugahara, 2013; Kuratani, Nobusada, Horigome, & Shigetani, 2001; Kuratani, Oisi, & Ota, 2016; Medeiros & Crump, 2012; Minarik et al., 2017; Myojin et al., 2001; Nikitina, Sauka‐Spengler, & Bronner‐Fraser, 2008; Oisi, Ota, Fujimoto, & Kuratani, 2013; Oisi, Ota, Kuraku, Fujimoto, & Kuratani, 2013; Olsson, Ericsson, & Cerny, 2005; Ota, Kuraku, & Kuratani, 2007; Sauka‐Spengler, Meulemans, Jones, & Bronner‐Fraser, 2007; Shigetani et al., 2002; Shone & Graham, 2014; Square, Jandzik, Romasek, Cerny, & Medeiros, 2017; Sugahara et al., 2011; Takio et al., 2004; Yao, Ohtani, Kuratani, & Wada, 2011). This level of conservation indicates that all vertebrates more or less deploy the same gene regulatory networks, signaling pathways, and developmental modules to specify their axes and determine the anatomical identity of the homologous structures from which their craniofacial complexes get built.

Epithelial–mesenchymal interactions also contribute to another generic aspect of pattern, which is the establishment of tissue type, and in particular the differentiation of craniofacial cartilage and bone (Balczerski et al., 2012; Bee & Thorogood, 1980; Cela et al., 2016; Couly et al., 2002; Dunlop & Hall, 1995; Ferguson et al., 2000; Francis‐West, Robson, & Evans, 2003; Hall, 1980, 1982, 1987; MacDonald & Hall, 2001; Merrill et al., 2008; Richman & Tickle, 1989; Richman & Tickle, 1992; Schowing, 1968; Shigetani, Nobusada, & Kuratani, 2000; Shigetani et al., 2002; Thorogood, 1987; Thorogood, Bee, & von der Mark, 1986; Tyler, 1978, 1983). For example one prominent hypothesis, originally deemed the “flypaper model” (Garrod, 1986; Thorogood, 1988, 1993) suggested that epithelial‐mesenchymal interactions promote the production of extracellular matrix, which acts as an adhesive that “traps” migrating neural crest cells at their site of differentiation and results in mesenchymal condensation and cartilage induction. Such epithelia would include the pharyngeal endoderm and surface ectoderm around the facial primordia, as well as the brain and sensory capsules, all of which have been shown to initiate and/or direct chondrogenesis at various stages of development (Hall, 1980, 1981, 2000, 2005; Hall & Miyake, 2000; Mina, Upholt, & Kollar, 1994; Miyake, Cameron, & Hall, 1996; Thorogood et al., 1986).

What has become apparent from the many types of experimental strategies undertaken and vertebrate models studied, is that the reciprocal interactions during craniofacial development between neural crest mesenchyme and surrounding epithelia are highly dynamic, hierarchical, and likely involve both cell‐autonomous and non‐autonomous signals (Noden & Schneider, 2006). But the penultimate morphological outcome for a majority of components throughout the craniofacial complex, especially in the jaws and face, arises as a function of the special intrinsic ability of neural crest cells to propagate species‐specific pattern by superimposing parameters like size and shape onto more generic aspects of pattern such as axial orientation, anatomical identity, and tissue type (Fish & Schneider, 2014b). This capacity has most likely enhanced the evolutionary plasticity and potential (i.e., adaptability) of structures that contain or rely upon neural crest derivatives (Donoghue, Graham, & Kelsh, 2008; Jheon & Schneider, 2009; Le Douarin et al., 2004; Schneider, 2005; Young et al., 2014) in the pharyngeal and rostral regions of the vertebrate head (Gans & Northcutt, 1983; Northcutt, 2005; Northcutt & Gans, 1983), the lateral wall of the mammalian skull (Schneider, 1999; Smith & Schneider, 1998), and during the process of domestication (Lord et al., 2016; Sanchez‐Villagra, Geiger, & Schneider, 2016; Wilkins, Wrangham, & Fitch, 2014).

In fact, as described below, the degree to which neural crest cells convey species‐specific patterning information, and the intrinsic mechanisms that they use, have been made most evident by leveraging chimeric transplant systems that exploit evolutionary differences among birds. Recent research in this area has begun to paint a clearer picture of how individual structures within the craniofacial complex acquire their species‐specific pattern, and notably, such work illustrates how developmental programs can become modified internally on the molecular and cellular levels so that morphological variation can be generated in a manner essential for evolution.

## 3 ORIGIN OF SPECIES‐SPECIFIC PATTERN AS REVEALED BY AVIAN CHIMERAS

Much like prior studies involving chimeras between different amphibian species, a well‐established and very useful experimental approach for investigating the developmental origins and patterning of craniofacial structures in amniote embryos has been the use of the quail‐chick chimeric system (Baker, Bronner‐Fraser, Le Douarin, & Teillet, 1997; Borue & Noden, 2004; Cobos, Shimamura, Rubenstein, Martinez, & Puelles, 2001; Couly & Le Douarin, 1990; Couly, Coltey, & Le Douarin, 1992; Couly, Coltey, & Le Douarin, 1993; Köntges & Lumsden, 1996; Le Douarin, 1973; Le Lièvre, 1978; Le Lièvre & Le Douarin, 1975; Noden, 1978b, 1983, 1986a; Noden & Schneider, 2006; Olivera‐Martinez, Coltey, Dhouailly, & Pourquie, 2000; Schneider, 1999; Schneider et al., 2001; Selleck & Bronner‐Fraser, 1995). Quail and chick are closely related birds with similar rates of growth and morphology (Ainsworth, Stanley, & Evans, 2010; Fitzgerald, 1969; Hamburger & Hamilton, 1951; Nakane & Tsudzuki, 1999; Padgett & Ivey, 1960; Smith et al., 2015; Zacchei, 1961). Surgical transplants between them have enabled the fates, functions, and behaviors of different cells and tissues to be observed throughout embryogenesis, and this has been indispensable to understanding countless facets of developmental biology (Abramyan & Richman, 2018; Le Douarin & McLaren, 1984; Le Douarin & Dieterlen, 2013; Le Douarin, Dieterlen‐Lievre, & Teillet, 1996; Noden, 1984; Noden & Schneider, 2006).

The success of the quail‐chick chimeric system stems from the fact that in general, avian embryos are easily accessible in ovo for all kinds of experimental manipulations. This includes grafting or extirpation of tissues through microsurgery, labeling of cells for lineage analysis, implantation of reagent‐soaked beads, injection of biochemicals, and manipulation of gene expression via retroviral infection or electroporation (Cerny, Lwigale, et al., 2004; Chen et al., 1999; Ealba et al., 2015; Eichele, Tickle, & Alberts, 1984; Fekete & Cepko, 1993; Fish & Schneider, 2014a; Fish et al., 2014; Hall et al., 2014; Johnston, 1966; Krull, 2004; Kulesa, Bronner‐Fraser, & Fraser, 2000; Larsen, Zeltser, & Lumsden, 2001; Logan & Tabin, 1998; Lwigale, Conrad, & Bronner‐Fraser, 2004; Lwigale, Cressy, & Bronner‐Fraser, 2005; Lwigale & Schneider, 2008; Momose et al., 1999; Nakamura & Funahashi, 2001; Noden, 1975; Schneider et al., 2001; Serbedzija, Bronner‐Fraser, & Fraser, 1989; Stocker, Brown, & Ciment, 1993; Woronowicz et al., 2018). One advantage of working in birds is that after surgery or other manipulations, eggs can simply be resealed and incubated until the embryos reach stages appropriate for further analysis.

Another factor contributing to the success of avian chimeric systems is that embryos from different avian species can be readily stage‐matched using an approach that relies on external morphological characters and that is independent of body size and incubation time (Hamilton, 1965; Ricklefs & Starck, 1998; Starck & Ricklefs, 1998). The Hamburger and Hamilton (HH) staging system, initially created for chick, is the accepted standard (Hamburger & Hamilton, 1951). Other staging systems have been published for quail (Ainsworth et al., 2010; Nakane & Tsudzuki, 1999; Padgett & Ivey, 1960; Zacchei, 1961) and duck (Koecke, 1958), but embryos of these birds can also be staged using the HH system for chicken (Ainsworth et al., 2010; Le Douarin et al., 1996; Lwigale & Schneider, 2008; Mitgutsch, Wimmer, Sanchez‐Villagra, Hahnloser, & Schneider, 2011; Schneider & Helms, 2003; Smith et al., 2015; Starck, 1989; Yamashita & Sohal, 1987; Young et al., 2014). The ease at which embryos of diverse types of birds can be stage‐matched has advanced the study of species‐specific patterning.

During her early and pioneering neural crest transplant work Le Douarin observed that, “Quail and chick cells, experimentally associated, definitively retain their species characteristics in the chimaera” (Le Douarin & Teillet, 1974, p. 163). Yet, while some examples related to patterning and pigmentation of epidermal appendages, as well as hatching behavior continued to be noted (Balaban, 1997; Balaban, Teillet, & Le Douarin, 1988; Sengel, 1990), for the most part because quail and chick are relatively similar, subtle species‐specific differences that may have been induced by donor cells have gone largely undetected. Moreover, determining mechanisms of species‐specific pattern was not really the primary goal of most studies that employed quail‐chick chimeras. In contrast, other experiments using avian chimeras have included domestic duck as a way to identify those patterning mechanisms that generate species‐specific differences (Dhouailly, 1967, 1970; Hampe, 1957; Lwigale & Schneider, 2008; Pautou, 1968; Schneider & Helms, 2003; Sohal, 1976; Sohal et al., 1990; Sohal et al., 1985; Tucker & Lumsden, 2004; Waddington, 1930; Waddington, 1932; Yamashita & Sohal, 1986, Yamashita & Sohal, 1987; Zwilling, 1959). Additional studies on species‐specific size and control of scaling have also included chimeras between quail and emu (Hall et al., 2014), quail and zebra finch (Chen, Balaban, & Jarvis, 2012), and chick and zebra finch (Uygur et al., 2016).

The quail‐duck chimeric transplant system has been especially useful for identifying the molecular and cellular basis for species‐specific aspects of pattern and for illuminating the mechanistic contributions of neural crest cells during tissue interactions that facilitate the structural and functional integration of the craniofacial complex (Figure 1b,c). The system itself combines classical grafting techniques and tools in vertebrate embryology that have already been mentioned (e.g., Andres, 1949; Hamburger, 1942; Harrison, 1917, 1921, 1924, 1929, 1935; Spemann, 1918, 1921, 1938; Spemann & Mangold, 1924; Spemann & Schotte, 1932; Twitty, 1934, 1945; Twitty & Schwind, 1931; Waddington, 1930, 1932; Wagner, 1959) with modern molecular and cellular methods and assays (Ealba & Schneider, 2013; Fish & Schneider, 2014a; Lwigale & Schneider, 2008). In short, presumptive neural crest cells from the midbrain and anterior hindbrain are transplanted from either quail to duck to create chimeric “quck” or from duck to quail to make chimeric “duail” (Ealba & Schneider, 2013; Fish & Schneider, 2014a; Lwigale & Schneider, 2008; Schneider & Helms, 2003) (Figure 1d). In this experimental framework, the ability to exploit chimeras between quail and duck embryos is predicated on three features that distinguish these species of birds.

First, quail and duck embryos and their constituent parts are noticeably different in size and shape, which offers a direct way to resolve if species‐specific features are mediated by donor‐ or host‐derived tissues. Second, quail and duck embryos develop at distinct rates (17 versus 28 days) (Figure 1e), which allows the effects of donor cells on the host to be readily assessed simply by looking for species‐specific changes to the timing of gene expression, tissue differentiation, and/or other events throughout the embryogenesis of the facial primordia (Figure 1f,g). Moreover, by examining the effects of intrinsic rates of maturation (i.e., differences in developmental time) on changes in morphology (i.e., evolutionary differences in size and shape), the quail‐duck chimeric system can help advance the study of the relationship between ontogeny and phylogeny, vis‐à‐vis the cranial neural crest (Schneider, 2018). Third, as is the case for the quail‐chick chimeric system (Le Douarin et al., 1996), there is an antibody (Q¢PN) that recognizes only quail cells, which permanently discriminates donor versus host derivatives (i.e., Q¢PN‐positive versus Q¢PN‐negative). Among other neural crest‐mediated outcomes, detection of this antibody enables species‐specific changes in gene expression patterns to be correlated with the distribution of donor cells in both donor‐ and host‐derived tissues (Figure 1h,i). Similarly, using species‐specific primers to amplify ubiquitously expressed housekeeping genes allows the ratio of quail versus duck cells, as well as any neural crest‐dependent changes to genes of interest, to be quantified on the molecular level using a PCR‐based strategy (Ealba & Schneider, 2013). Therefore, as designed, the quail‐duck chimeric system permits the role of the neural crest (and other cell populations) to be characterized during development and provides a powerful tool for ascertaining precisely when and where morphogenetic events and gene regulatory networks become modified as a means to generate species‐specific pattern in the craniofacial complex.

## 4 ORIGIN OF SPECIES‐SPECIFIC PATTERN IN THE CRANIOFACIAL INTEGUMENT

Like what has been described earlier for studies on the pigmentation of amphibians, the ability of neural crest cells to mediate species‐specific pattern is most readily apparent in the integument of quail‐duck chimeras (Eames & Schneider, 2005; Schneider, 2005; Schneider & Helms, 2003). Moreover, in many ways the integument can serve as a microcosm for understanding how reciprocal epithelial‐mesenchymal interactions drive the patterning of a broad range of vertebrate organ systems including the limbs, facial primordia, hair, glands, teeth, and bone (Dunlop & Hall, 1995; Fisher, 1987; Francis‐West et al., 1998; Hu et al., 2003; Hughes et al., 2018; Lumsden, 1988; Mitsiadis, Hirsinger, Lendahl, & Goridis, 1998; Pispa & Thesleff, 2003; Richman & Tickle, 1992; Salaun, Salzgeber, & Guenet, 1986; Saunders & Gasseling, 1968; Schneider et al., 1999; Schneider et al., 2001; Sharpe & Ferguson, 1988; Shigetani et al., 2000; Thesleff & Sharpe, 1997; Tonegawa, 1973; Tucker, Al Khamis, & Sharpe, 1998; Wang, Upholt, Sharpe, Kollar, & Mina, 1998; Wedden, 1987). The integument is composed partly of epidermis, which is derived from the non‐neural ectoderm and is stratified into multiple layers (Hamilton, 1965; Romanoff, 1960; Yasui & Hayashi, 1967). In amniotes, the uppermost layer of epidermis generally produces the keratinized components associated with epidermal appendages such as feathers, scales, hair, horns, beaks, and egg teeth (Couly & Le Douarin, 1988; Kingsbury, Allen, & Rotheram, 1953; Lucas & Stettenheim, 1972; Pera et al., 1999; Pispa & Thesleff, 2003; Sawyer, O'Guin, & Knapp, 1984; Yu et al., 2004).

**Figure 2:**
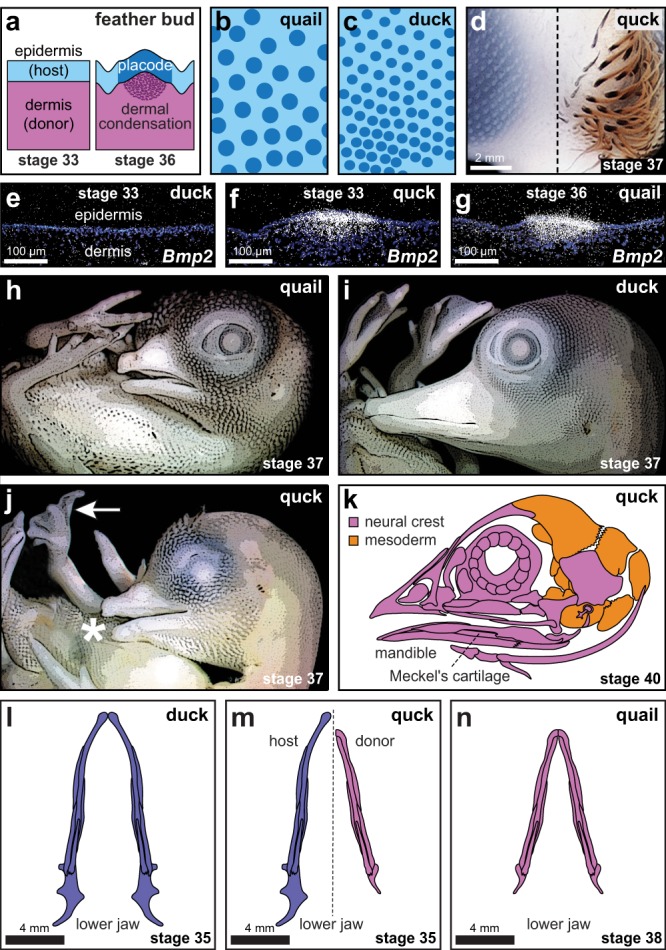
**(a)** Cranial feather buds form through interactions between neural crest‐derived dermis and overlying epidermis, which in chimeras are derived from donor and host, respectively. At stage 33, there is little evidence for feather development, but by stage 36, feather buds contain dermal condensations and they begin to rise above the surface of the integument. **(b)** Quail cranial feather buds are large and widely spaced whereas those of **(c)** duck are smaller and more closely spaced. **(d)** In chimeric quck, quail‐like feathers appear at the long bud stage while those derived from the duck host are still short buds. **(e)** At stage 33, *Bmp2* is not expressed in either the dermis or epidermis. However, in **(f)** chimeric quck at stage 33 *Bmp2* is expressed prematurely in donor‐derived dermis as well as in host‐derived epidermis like what is observed three stages later in **(g)** control quail (and duck). Modified from Schneider (2005), Eames and Schneider (2005), and Fish and Schneider (2014b). **(h)** The beaks of quail embryos are short and blunt whereas those of **(i)** duck are long and broad. **(j)** Transplants of presumptive cranial neural crest cells, which are destined to form the beak, from quail donors to duck hosts produce chimeric “quck” embryos with quail‐like beak size and shape (asterisk). Note that the quail‐like quck has webbed feet (arrow), which is indicative of the duck host. Modified from Schneider (2005). **(k)** The extent of transformation in chimeras corresponds to the boundary that exists in the skull between those bones and cartilages derived from the cranial neural crest and those formed from mesoderm. Based on a drawing from D. Noden. **(l)** By stage 35, Meckel's cartilage and the lower jaw are slightly curved in duck as seen in dorsal view. **(m)** This curved morphology is maintained on the duck host side of chimeric quck whereas Meckel's cartilage and the lower jaw appear to straighten out on the donor side and achieve a larger size like that observed in a **(n)** quail embryo three stages later at stage 38. Modified from Fish and Schneider (2014b)

Beneath the epidermis lies the dermis, which in the trunk and posterior regions of the head comes from mesodermal mesenchyme, whereas in the face and aspects of the neck the dermis originates from neural crest mesenchyme (Couly et al., 1992; Matsuoka et al., 2005; Noden, 1978b, 1986b, 1988; Olivera‐Martinez et al., 2000; Olivera‐Martinez, Thelu, & Dhouailly, 2004). As discussed already, neural crest cells also generate the pigment‐producing melanocytes that infiltrate the epidermis and supply color to the skin and epidermal appendages (Bronner‐Fraser, 1994; Cramer, 1991; Hirobe, 1995; Le Douarin & Dupin, 1993; Noden, 1978a; Rawles, 1944, 1948). Patterning and differentiation of the integument relies upon a series of reciprocal signaling interactions between the mesenchyme of the dermis and the epithelium of the epidermis (Dhouailly, 1973, 1975; Lucas & Stettenheim, 1972; Pispa & Thesleff, 2003; Widelitz & Chuong, 1999; Widelitz et al., 1997). Feather development starts when mesenchyme aggregates into a thin layer called “dense dermis” beneath the epithelium (Brotman, 1977; Mayerson & Fallon, 1985; Wessells, 1965). Subsequently, the overlying epithelium thickens into a series of epidermal placodes, the dense dermis forms discrete mesenchymal condensations, the mesenchyme and placodes emerge out of the surface of the integument as feather buds (Figure 2a), and both tissues grow and differentiate into discrete feathers (Olivera‐Martinez, Viallet, Michon, Pearton, & Dhouailly, 2004; Pispa & Thesleff, 2003). Together, feathers form in continuous rows that make up tracts (Lucas & Stettenheim, 1972).

The timing of induction as well as species‐specific characteristics such as the location, number, distribution, pigmentation, and size of epidermal appendages like feathers is determined by dermis (Cairns & Saunders, 1954; Dhouailly, 1967, 1970, 1973; Dhouailly & Sawyer, 1984; Eames & Schneider, 2005; Fliniaux, Viallet, & Dhouailly, 2004; Linsenmayer, 1972; Prin & Dhouailly, 2004; Rawles, 1963; Saunders & Gasseling, 1957; Schneider, 2005; Song & Sawyer, 1996; Wessells, 1965). In other instances and depending on the stage of development, the epithelium can provide patterning information on anatomical identity such as specifying whether epidermis produces feathers or scales (Chuong, Chodankar, Widelitz, & Jiang, 2000; Prin & Dhouailly, 2004; Rawles, 1963; Widelitz, Jiang, Lu, & Chuong, 2000) or in controlling late‐stage branching patterns (Harris, Fallon, & Prum, 2002; Yu, Wu, Widelitz, & Chuong, 2002). But both tissues collaborate to form feathers. For instance, when dermis and epidermis from different‐staged wild type and featherless chicken mutants are recombined, the dermis is able to induce epidermal placodes, although this ability goes away quickly without proper epidermal interactions (Viallet et al., 1998). The dominant role of the dermis can be most clearly appreciated using quail‐duck chimeras.

The embryos of Japanese quail (*Coturnix coturnix japonica*) have large, widely spaced, and pigmented feather buds whereas the embryos of white Pekin duck *(Anas platyrhynchos)* have relatively smaller, tightly arranged, and un‐pigmented feather buds (Lucas & Stettenheim, 1972; Schneider, 2005) (Figure 2b,c). In quail‐duck chimeras, where quail donor neural crest cells generate the dermis and duck host ectoderm gives rise to the epidermis, cranial feather pattern acquires the identity of the donor species (Eames & Schneider, 2005). Coincident with the distribution of quail donor neural crest‐derived dermis, these chimeric “quck” contain long brown and black quail‐like feathers assembled among short white duck host feather buds (Figure 2d). Conversely, when duck donor neural crest cells are transplanted into quail hosts, the chimeric “duail” embryos have unpigmented duck‐like feathers. Thus, quail‐duck chimeras corroborate the role of the neural crest (and by extension the dermis throughout the integument) as the principal source of species‐specific patterning information for cranial feathers. These results align with data from other tissue recombination experiments in the trunk between duck and chick, which also indicated that the dermis was a source of species‐specific pattern for the feathers (Dhouailly, 1967, 1970). But exactly how does neural crest‐derived dermis impose its species‐specific will on the epidermis?

To accomplish this task, neural crest‐derived dermis executes an autonomous molecular feather program that is not only intrinsic to the donor genome, but also that overrides the epidermal feather program of the host. This phenomenon becomes most readily apparent when examining changes to the spatial and temporal domains of gene expression, and rates of feather bud development in quck and duail chimeras (Eames & Schneider, 2005). In particular, donor neural crest modifies the expression of members and targets of the SHH, BMP, and Delta/Notch pathways, which are well known to regulate normal feather morphogenesis (Ashique, Fu, & Richman, 2002b; Chuong et al., 2001; Chuong, Patel, Lin, Jung, & Widelitz, 2000; Crowe, Henrique, Ish‐Horowicz, & Niswander, 1998; Morgan, Orkin, Noramly, & Perez, 1998; Patel, Makarenkova, & Jung, 1999; Pispa & Thesleff, 2003; Ting‐Berreth & Chuong, 1996; Widelitz et al., 1997; Yu et al., 2002). In chimeras, each of these signaling pathways shows a significant change in the timing and domains of expression in both the dermis and epidermis corresponding to the species and stage of the donor neural crest.

For example, *Bmp2* is one of the earliest genes to be expressed wherever the epidermis begins to thicken into placodes along the presumptive feather tracts as well as in the underlying condensations of dermis; likewise, *Bmp4* expression is restricted to the dermis as the mesenchyme begins to condense and thereafter (Chuong, Widelitz, Ting‐Berreth, & Jiang, 1996; Jung et al., 1998; Nohno et al., 1995; Noramly & Morgan, 1998; Scaal et al., 2002; Widelitz et al., 1997). In chimeric quck, the timing of *Bmp2* and *Bmp4* expression is accelerated by three stages not only in the faster‐developing quail donor‐derived dermis, but also *Bmp2* is expressed prematurely in the normally slower‐developing duck host‐derived epidermis (Figure 2e–g). The same is observed for all other genes examined, which become spatially and temporally regulated by donor dermis. In other words, the dermis specifies the pattern through which epidermis forms feather buds by adhering to the timetable of the donor species and by defining the expression domains of members and targets of the SHH, BMP, and Delta/Notch pathways (Eames & Schneider, 2005).

This remarkable capacity holds true in reverse as evidenced by duail chimeras where slower‐developing duck dermis acts out its feather program on a delayed timetable relative to quail host epidermis and produces duck‐like feathers. Extrapolating these results, we would predict that the dermis (of both neural crest and mesodermal origin) most likely regulates in a species‐specific manner many other genes known to function during feather morphogenesis such as members and targets of the FGF, Epidermal Growth Factor, and WNT pathways (Atit, Conlon, & Niswander, 2003; Chang et al., 2004; Chodankar et al., 2003; Mandler & Neubuser, 2004; Noji et al., 1993; Noramly, Freeman, & Morgan, 1999; Olivera‐Martinez, Thelu, Teillet, & Dhouailly, 2001; Rouzankina, Abate‐Shen, & Niswander, 2004; Song, Wang, & Goetinck, 1996; Song, Lee, & Goetinck, 2004; Tanda, Ohuchi, Yoshioka, Noji, & Nohno, 1995; Tao et al., 2002; Widelitz, Jiang, Chen, Stott, & Chuong, 1999; Widelitz, Jiang, Noveen, Chen, & Chuong, 1996). Thus, neural crest cells exert their species‐specific will by presiding at the top of hierarchical conversations with their neighbors (e.g., epidermis), autonomously implementing their own molecular and cellular agendas, and dictating the terms of morphogenetic events in space and time.

## 5 ORIGIN OF SPECIES‐SPECIFIC PATTERN IN THE BEAK

As is the case for the integument, quail‐duck transplants likewise demonstrate that neural crest cells provide species‐specific information for patterning the beak and underlying jaw skeleton, which differ substantially between quail and duck in conjunction with their highly specialized modes of feeding (Figure 2h,i). Quail neural crest cells destined to form the beak skeleton make quail‐like beaks on duck hosts and reciprocal transplants of duck neural crest cells generate duck‐like bills on quail hosts (Schneider & Helms, 2003) (Figure 2j,k). Equivalent species‐specific transformations are observed when neural crest cells fated to become cartilages in the jaw joint are transplanted between quail and duck (Tucker & Lumsden, 2004). Overall, these studies using quail‐duck chimeras reinforce the key role for neural crest in establishing species‐specific morphology of the beak and jaw apparatus.

However, such results are not really surprising given that the jaw skeleton is derived entirely from the neural crest (Couly et al., 1993; Köntges & Lumsden, 1996; Le Lièvre, 1978; Le Lièvre & Le Douarin, 1975; Noden, 1978b), and also because the long history of chimeric grafting experiments discussed earlier had already revealed the special species‐specific properties of this lineage. But gaining the ability to distinguish between beak tissues that arise from the donor versus the host with a high degree of certainty, as well as possessing tools to assay for donor‐mediated changes in gene expression, is what sets this modern chimeric strategy apart from earlier studies (Ealba & Schneider, 2013; Fish & Schneider, 2014a; Lwigale & Schneider, 2008; Schneider & Helms, 2003). A first critical insight in this regard came from examining changes to beak tissues derived from the host. For instance, at the tip of their bill, duck have an egg tooth that is a flat epidermal nail, whereas quail develop an egg tooth that is a conical protrusion of hard keratin (Lucas & Stettenheim, 1972). The quck egg tooth, despite arising entirely from non‐transplanted duck host epidermis, resembles that found in quail. Similarly, the duail egg tooth looks like that of the duck. This clear transfer of patterning information from donor neural crest to non‐neural crest host‐derived tissues reveals that the transformation of the beak in chimeras is more‐or‐less comprehensive and helps explain how the beak can become modified as an integrated morphological unit in its entirety during the course of evolution (Schneider, 2005).

A second important discovery arose after analyzing genes that are known to pattern the face and that also show well‐defined periods of expression during development. Because quail and duck have distinct rates of maturation, the initiation and cessation of expression of such genes differ in absolute time. For example, twenty‐four hours after surgery, control quail embryos express the transcription factors *Barx1* and *Msx1* in neural crest‐derived mesenchyme of the developing beak primordia, but control duck embryos do not yet express these genes because they require a longer period of time to reach an equivalent stage. Correspondingly and quite strikingly in quck chimeras, these genes are expressed in mesenchyme derived from the quail donor but not from the duck host. Similarly, 48 hr after surgery, control quail express *Shh* but not *Pax6* in facial ectoderm whereas control duck express *Pax6* but not *Shh.* In quck chimeras, *Shh* is found in duck host facial ectoderm but *Pax6* is not, which is the pattern observed in quails. As already discussed, *Shh* expression in the facial ectoderm for instance, is not only mediated by the neural crest (Hu & Marcucio, 2012; Schneider et al., 2001), but also plays a critical role in specifying the axial orientation and maintaining the outgrowth of the facial skeleton (Abzhanov & Tabin, 2004; Ahlgren & Bronner‐Fraser, 1999; Chong et al., 2012; Delloye‐Bourgeois et al., 2014; Helms et al., 1997; Hu & Helms, 1999; Hu & Marcucio, 2009a; Hu, Young, Li, et al., 2015; Jeong, Mao, Tenzen, Kottmann, & McMahon, 2004; Lan & Jiang, 2009; Young et al., 2010). Thus, such temporal shifts in the onsets and offsets of gene expression supply stark evidence that quail neural crest cells produce quail‐like beaks on duck by sustaining their own molecular programs and by modulating the spatial and temporal patterns of gene expression in non‐neural crest host tissues such as the adjacent epithelium.

Again, as noted by Raven (1933) and conveyed by Hörstadius (1950), part of the special ability of neural crest to regulate species‐specific pattern likely arises “in connection with its movements, as a carrier of inductive influences” (p. 93). Simple parameters such as the amount and distribution of donor neural crest cells throughout the facial primordia appears to modulate gene expression in host epithelium in a dose‐dependent manner (Ealba & Schneider, 2013; Merrill et al., 2008; Schneider & Helms, 2003). Once a certain threshold is reached (Woronowicz et al., 2018), neural crest cells ultimately endow structures with species‐specific size and shape presumably because their “inductive influences” are mediated at the population level. This scenario is also substantiated by our observation that significant differences exist in the number of jaw precursors that migrate into the mandibular primordia of duck versus quail (Fish & Schneider, 2014c; Fish et al., 2014). During neurulation, duck generate about 15% more pre‐migratory neural crest cells at the levels of the midbrain and rostral hindbrain, and these are the cells that will ultimately enable them to build their long bills. Only a few stages later, duck have twice as many cells in their mandibular primordia as do quail due to specific‐specific variation in cell proliferation dynamics and cell cycle length.

Cell cycle length in duck mandibular mesenchyme is longer (13.5 hr) than in quail (11 hr), and this might seem counterintuitive given that duck make more cells, but when the total duration of each embryonic stage during this developmental window is considered in terms of absolute time (i.e., 45 hr for duck versus 32 hr for quail), then duck cells wind up proliferating more in total than those of quail. Therefore, by sustaining their slower intrinsic maturation rate over a longer period of time, duck implement a cellular mechanism that progressively increases jaw size during development (Fish & Schneider, 2014b; Fish et al., 2014; Schneider, 2015, 2018). In this way, duck seem to rely on time as one means to control size, which supports prior observations in birds on the correlation between innate rates of growth and body size (Ricklefs & Starck, 1998; Starck, 1989; Starck & Ricklefs, 1998).

## 6 ORIGIN OF SPECIES‐SPECIFIC PATTERN IN THE CARTILAGINOUS SKELETON

To explain more precisely how differences in the rates of development and the numbers of neural crest cells that get allocated to the mandibular primordia become species‐specific determinants of size and shape, we focused on the differentiation and growth of Meckel's cartilage in the lower jaw skeleton (Eames & Schneider, 2008). Meckel's cartilage develops from neural crest mesenchyme into a cylindrical rod that rarely ever ossifies except in the proximal‐most region(De Beer, 1937; Eames, Sharpe, & Helms, 2004; Ekanayake & Hall, 1994; Helms & Schneider, 2003; Kavumpurath & Hall, 1990; Noden, 1978b). During cartilage formation, pre‐chondrogenic cells first undergo condensation and then begin overt differentiation, where they secrete extracellular matrix (Eames, de la Fuente, & Helms, 2003; Hall, 2005). There is a well‐documented relationship between condensation size and skeletal size (Hall & Miyake, 1992, 1995, 2000; Miyake et al., 1996; Smith & Schneider, 1998), and from the earliest stages of chondrogenesis, we observe smaller condensations in quail relative to duck (Eames & Schneider, 2008). When we transplant presumptive neural crest from quail embryos into stage‐matched duck we do so unilaterally (Figure 1d), which allows these quail donor neural crest cells to fill one side of the duck host mandible and enables us to compare the development of donor quail‐derived versus host duck‐derived Meckel's cartilage in the same chimeric quck. While the sequential stages of chondrogenesis are comparable in quail and duck, in quck chimeras, we find that quail donor neural crest cells make smaller condensations and differentiate into cartilage on a faster timetable (i.e., three stages ahead of the duck).

Accompanying these changes in quck chimeras is the premature expression of chondrogenic genes by quail donor cells relative to duck host cells on the contralateral side. For example, *Sox9*, which is an early molecular marker of chondrogenic condensations (Eames et al., 2003; Eames et al., 2004; Healy, Uwanogho, & Sharpe, 1996; Zhao, Eberspaecher, Lefebvre, & De Crombrugghe, 1997), and *Col2a1*, which is directly regulated by *Sox9* (Bell et al., 1997) are both upregulated coincident with the presence of quail donor neural crest mesenchyme. Additionally, we find that FGF signaling, which operates upstream of *Sox9* and chondrogenesis (Bobick, Thornhill, & Kulyk, 2007; De Crombrugghe et al., 2000; Eames et al., 2004; Govindarajan & Overbeek, 2006; Healy, Uwanogho, & Sharpe, 1999; Murakami, Kan, McKeehan, & de Crombrugghe, 2000; Petiot, Ferretti, Copp, & Chan, 2002) is also regulated by neural crest mesenchyme as evidenced by analyzing expression of the ligands *Fgf4* and *Fgf8*, and the receptor *Fgfr2.* While FGF ligands are known to be expressed continuously in mandibular epithelium from the earliest embryonic stages onward (Havens, Rodgers, & Mina, 2006; Mina et al., 2002; Shigetani et al., 2000; Wall & Hogan, 1995), we find that in chimeras the receptor *Fgfr2* is expressed three stages earlier by quail donor neural crest mesenchyme. If FGF signaling is blocked during this discrete temporal window when *Fgfr2* becomes activated, then Meckel's cartilage fails to form.

Ultimately, by exerting control over the timing of FGF signaling and the expression of downstream targets such as *Sox9* and *Col2a1*, neural crest mesenchyme likely provides cues on the molecular level that impart Meckel's cartilage with species‐specific size and shape. Such a conclusion is also based on our observation that on the morphological level Meckel's cartilage displays obvious stage‐specific and species‐specific differences in size and shape throughout development in quail and duck, and that these differences are maintained by quail donor neural crest mesenchyme in quck chimeras (Figure 2l–n). For example, Meckel's initially forms in both duck and quail as a slightly curved cartilage that becomes more S‐shaped. Shortly thereafter, Meckel's in duck remains curved while Meckel's in quail straightens out. Meckel's continues to grow in both quail and duck, but increasingly gets larger in duck. In quck chimeras, quail donor neural crest maintains its faster maturation rate within the relatively slower duck host, and the differentiation of Meckel's cartilage gets accelerated by approximately three stages on the donor side. Furthermore, the size and shape of Meckel's cartilage on the donor side becomes consistently more quail‐like compared with that observed on the contralateral duck host side.

So, while surrounding endodermal and ectodermal epithelia seem to define “where” cartilage condensations form along an axis, which is most likely equivalent between quail and duck, our chimeric transplant experiments reveal that neural crest defines “when” and “what” by responding through intrinsic programs that control both stage‐specific and species‐specific size and shape. Significantly, this ability to keep track of stage‐specific and species‐specific size and shape simultaneously indicates that the neural crest cells themselves can function as a potent mechanism linking ontogeny and phylogeny. Such were the predictions made by proponents of heterochrony who argued that changes in the timing of developmental events and/or rates of growth had direct implications for the evolution of size and shape (Alberch et al., 1979; De Beer, 1930; Foster & Kaesler, 1988; Gould, 1977; Hall, 1984; Klingenberg & Spence, 1993; McKinney, 1988; Raff, 1996; Roth, 1984; Russell, 1916; Schneider, 2018). In this regard, faster‐developing quail donor neural crest mesenchyme not only induces a heterochrony by altering the rates of growth in chimeras, but also, the presence of these cells appears to introduce shifts in the relative onsets, cessations, or durations of molecular and cellular events, which is an additional process through which changes in time can affect size and shape (Smith, 2001, 2002, 2003), especially in the context of reciprocal epithelial–mesenchymal interactions underlying skeletal evolution (Smith & Hall, 1990). Our transplants in birds with even larger disparities in growth rates like quail and emu (i.e., 17 versus 58 days from fertilization to hatching), which are separated by about seven embryonic stages during chondrogenesis, reveal that there are very few developmental constraints that prevent the host from supporting the execution of neural crest mesenchyme‐dependent programs for skeletogenesis (Hall et al., 2014).

## 7 ORIGIN OF SPECIES‐SPECIFIC PATTERN IN THE BONY SKELETON

Akin to what we have found with cartilage, neural crest mesenchyme likewise communicates species‐specific information on size and shape to bone in the jaws and facial skeleton by establishing the timing of major events during osteogenesis. In quck chimeras, quail donor neural crest mesenchyme upholds its faster timetable for maturation and autonomously executes molecular and cellular programs that promote and orchestrate each individual step of osteogenesis including the induction, proliferation, differentiation, mineralization, and remodeling of bone (Ealba et al., 2015; Hall et al., 2014; Merrill et al., 2008). Such a capacity by neural crest mesenchyme to function as a developmental timekeeper supports earlier theoretical predictions (Alberch, 1982a; Oster & Alberch, 1982; Oster et al., 1988) about how quantitative changes to parameters within ontogenetic systems can drive morphological evolution (Schneider, 2018). For example, genetic or epigenetic modifications to “biochemical, cell–cell, or tissue interactions” (Alberch, 1985, p. 50) can in turn alter “rates of diffusion, mitotic rate, cell adhesion, etc.” (Alberch, 1989, p. 27), which can then cause evolutionary changes in size and shape. Similarly, our work reveals that such quantitative changes to cellular parameters like neural crest‐mediated differences between quail and duck in the number of progenitors, rate of proliferation, length of the cell cycle, and timing of differentiation leads to morphological outcomes in the bony skeleton that are species‐specific.

Along these lines and as a case in point, we find that neural crest mesenchyme establishes the timing of osteogenesis in the jaw by regulating cell cycle progression (Hall et al., 2014). Seemingly, neural crest mesenchyme controls the cell cycle through stage‐ and species‐specific expression of cyclin and cyclin‐dependent kinase inhibitors (CKI) including *p27* (*cdkn1b*), which is a CKI that can decrease proliferation in differentiating osteoblasts; *Cyclin E* (*Ccne1*), which is needed for G1/S phase transition; and *Cyclin B1* (*Ccnb1*), which is essential for G2/M phase transition (Coats, Flanagan, Nourse, & Roberts, 1996; Drissi et al., 1999; Zavitz & Zipursky, 1997). We find species‐specific differences in the expression and post‐translational processing of these cell cycle regulators, which we predict could permit birds like quail to shorten their period of mesenchymal proliferation and lead to faster‐differentiating and smaller beak skeletons. For example, in quail and on the donor side of quck, we find that *p27* is up‐regulated relative to that observed in duck. Previous experiments have demonstrated that *p27* is associated with size, including *p27*‐deficient mice, which are much larger than their wild‐type littermates yet have no obvious defects in their skeletons (Drissi et al., 1999). Likewise, the frontonasal process of duck has lower *p27* levels than that observed in chick (Powder et al., 2012), and furthermore the mandibular primordia shows tissue‐specific post‐translational regulation of *p27*, like what has been reported in other systems (Hirano et al., 2001; Zhang, Bergamaschi, Jin, & Lu, 2005). Therefore, changing *p27* levels may affect tissue‐ and species‐specific size and/or total growth. This direct connection between the regulation of cell cycle progression and the timing of events throughout bone development offers a mechanism through which neural crest mesenchyme might be able to generate changes in skeletal size and shape during evolution.

The potential for such a mechanism is further supported by experiments in which we can mimic the results observed in quck chimeras by prematurely inducing cell cycle exit. In this scenario, we find that the molecular program for osteogenesis becomes accelerated (Hall et al., 2014). Specifically, we observe early and elevated expression of genes such as *Runx2*, which is known to be a “master regulator” of bone formation that can direct osteoblast differentiation, influence skeletal size, and control the timing of mineralization (Ducy et al., 1999; Ducy, Zhang, Geoffroy, Ridall, & Karsenty, 1997; Eames et al., 2004; Galindo et al., 2005; Komori et al., 1997; Maeno et al., 2011; Otto et al., 1997; Pratap et al., 2003; Thomas et al., 2004). Neural crest mesenchyme in the mandibular primordia normally expresses *Runx2* during a tightly controlled temporal window and at well‐defined levels (Eames et al., 2004; Merrill et al., 2008), but by over‐expressing *Runx2* prematurely and at higher levels in chick embryos, we can markedly reduce the size of the beak skeleton (Hall et al., 2014). This in effect, reflects the relationship normally observed between endogenous *Runx2* levels and species‐specific beak size. In fact, when the quail jaw skeleton begins to mineralize, its *Runx2* levels are more than twice that found in duck. Along these lines, other studies have hypothesized that there is a mechanistic link between predicted differential levels of *Runx2* expression (based on ratios of tandem repeats in DNA) and the length of the face among dogs and other mammals (Fondon and Garner, 2004; Pointer et al., 2012; Sears, Goswami, Flynn, & Niswander, 2007).

That neural crest mesenchyme regulates the timing and levels of *Runx2* expression, and that this in turn has a direct effect on skeletal size, fulfills predictions made more than 75 years ago by embryologists such as Huxley (1932) and Goldschmidt (1938, 1940) with regard to the existence of genes that establish the time and rate of development (Schneider, 2018). Along similar lines, De Beer (1954) argued that, “by acting at different rates, the genes can alter the time at which certain structures appear” (p. 20). Data from in vitro experiments also help explain how *Runx2* could function in this capacity whereby *Runx2* expression both depends upon and regulates cell cycle progression through mechanisms such as the repression of rRNA synthesis and the up‐regulation of *p27* (Galindo et al., 2005; Pratap et al., 2003; Thomas et al., 2004; Young et al., 2007). Collectively, these observations indicate that neural crest mesenchyme establishes species‐specific size and shape in the bony skeleton by mediating the timing of the transition from proliferation to differentiation and by modulating the expression levels of osteogenic transcription factors such as *Runx2.*

Neural crest mesenchyme also appears to exert control over species‐specific size and shape during osteogenesis upstream of *Runx2* by governing the temporal and spatial expression of members and targets of the BMP pathway (Merrill et al., 2008). BMP ligands can induce bone formation both embryonically (Kingsley et al., 1992; Luo et al., 1995; Solloway et al., 1998) and postnatally (Urist, 1965; Wang et al., 1990; Wozney et al., 1988). During jaw development, *Bmp2*, *Bmp4*, and *Bmp7*, as well as their receptors (*Bmpr1a*, *Bmpr1b*, and *Alk2*) are expressed in mandibular mesenchyme and/or epithelium (Ashique et al., 2002a; Bennett, Hunt, & Thorogood, 1995; Francis‐West, Tatla, & Brickell, 1994; Wall & Hogan, 1995), and they play critical roles during osteogenesis (Ashique et al., 2002b; Francis‐West et al., 1998; Wang et al., 1998). For example, BMP4 helps neural crest mesenchyme differentiate into bone (Abzhanov, Rodda, McMahon, & Tabin, 2007) and the lower jaw fails to form when *Bmp4* is conditionally eliminated from mandibular epithelium (Liu et al., 2005). BMP signaling regulates osteogenesis via a highly conserved pathway (Derynck, Piek, Schneider, Choy, & Alliston, 2008; Heldin, Miyazono, & ten Dijke, 1997; Kawabata, Imamura, & Miyazono, 1998; Massague & Wotton, 2000) involving *Smad* activation, which in turn affects *Runx2* expression (Ducy, 2000; Ducy et al., 1997; Kang, Alliston, Delston, & Derynck, 2005; Karsenty et al., 1999; Komori et al., 1997) and mandibular osteogenesis (Otto et al., 1997). Moreover, physical interactions between SMAD proteins and *Runx2* drive osteoblast‐specific gene expression (Alliston, Choy, Ducy, Karsenty, & Derynck, 2001; Ito et al., 2002; Lee et al., 2000). Other targets of BMP signaling including *Msx1* (Tribulo, Aybar, Nguyen, Mullins, & Mayor, 2003) play a role during the epithelial–mesenchymal interactions of the mandible (Bei & Maas, 1998; Chen & Struhl, 1996; Han et al., 2007), are neural crest‐mediated (Schneider & Helms, 2003), and affect bone formation (Roybal et al., 2010; Satokata & Maas, 1994).

As part of its osteo‐inductive role, BMP signaling likely shapes the avian beak by creating domains of differential growth within the mesenchyme. For instance, distinct domains of *Bmp4* expression in the frontonasal primordium contribute to beak width and depth among birds including Darwin's finches, cockatiels, chicks, and ducks (Abzhanov et al., 2004; Schneider, 2007; Wu et al., 2006, 2004). Likewise, over‐expressing *Bmp4* in cichlid fish that usually form elongated jaws, shortens and widens the jaw and in effect phenocopies features that are coupled with the evolution of distinct feeding strategies (Albertson, Streelman, Kocher, & Yelick, 2005). This salient ability of neural crest mesenchyme to control the timing of osteogenesis by autonomously executing molecular programs involving BMP signaling as well as transcriptional targets such as *Msx1* and *Runx2*, likely serves as a key developmental mechanism facilitating the evolution of species‐specific size and shape in the craniofacial skeleton.

## 8 ORIGIN OF SPECIES‐SPECIFIC PATTERN DURING BONE RESORPTION

While much of the work we have performed has demonstrated that neural crest mesenchyme conveys species‐specific size and shape to the craniofacial skeleton by regulating molecular and cellular programs for the induction and deposition of cartilage and bone, we have also discovered that a previously underappreciated but potentially just as important mechanism affecting species‐specific size and shape lies in the ability of neural crest mesenchyme to direct the process of bone resorption (Ealba et al., 2015; Schneider, 2015). Usually, bone resorption is tied to bone deposition as a metabolic function for maintaining homeostasis in the adult skeleton (Buckwalter, Glimcher, Cooper, & Recker, 1996; Filvaroff & Derynck, 1998; Hall, 2005; Nguyen, Tang, Nguyen, & Alliston, 2013; O'Brien et al., 2008; Teitelbaum, 2000; Teitelbaum, Tondravi, & Ross, 1997). In contrast, little is known about the role of resorption during skeletal patterning in embryos, except for a few hypotheses about the effects of differential fields of bone resorption on the size and shape of the developing human jaw skeleton (Enlow, Moyers, & Merow, 1975; Moore, 1981; Radlanski & Klarkowski, 2001; Radlanski, Renz, Lajvardi, & Schneider, 2004) and recent work on the remodeling of Meckel's cartilage by chondroclasts in mammals (Anthwal, Urban, Luo, Sears, & Tucker, 2017).

When we assay for molecular and enzymatic markers of bone resorption we observe significantly higher levels and distinct spatial domains in quail versus duck that correlate with species‐specific differences in beak size and shape. There are two populations of cells that resorb bone in the craniofacial skeleton. Osteoclasts arise from the mesodermal hematopoietic lineage (Couly, Coltey, Eichmann, & Le Douarin, 1995; Couly et al., 1992; Jotereau & Le Douarin, 1978; Kahn et al., 2009), and osteocytes (Akil et al., 2014; Belanger, 1969; Fowler et al., 2017; Jauregui et al., 2016; O'Brien et al., 2008; Qing et al., 2012; Tang, Herber, Ho, & Alliston, 2012; Xiong & O'Brien, 2012; Xiong et al., 2014) are derived entirely from neural crest mesenchyme (Helms & Schneider, 2003; Le Lièvre, 1978; Noden, 1978b). Therefore, in our quail‐duck chimeras, all osteoclasts form exclusively from the host mesoderm whereas all osteocytes come from the donor neural crest.

Osteoclasts and osteocytes secrete tartrate‐resistant acid phosphatase (TRAP) when they are actively resorbing bone (Minkin, 1982; Qing et al., 2012; Tang et al., 2012). Also, osteoclasts express *Matrix Metalloproteinase 9* (*Mmp9)* (Engsig et al., 2000; Reponen, Sahlberg, Munaut, Thesleff, & Tryggvason, 1994) and osteocytes express *Mmp13* (Behonick et al., 2007; Johansson et al., 1997; Sasano et al., 2002). Accordingly, in the lower jaw of chimeric quck, *Mmp9* is expressed by duck host‐derived osteoclasts while *Mmp13* is expressed by quail donor‐derived osteocytes. We find that control quail express substantially higher levels of TRAP, *Mmp9*, and *Mmp13* than do duck, suggesting that increased resorption may relate to shorter beaks (Ealba et al., 2015). Similarly, chimeric quck have greatly elevated TRAP, *Mmp9*, and *Mmp13* expression in association with the donor‐mediated transformation into quail‐like beaks, which implies that quail donor neural crest mesenchyme executes an autonomous species‐specific program that controls bone resorption by its own derivatives (i.e., osteoclasts) as well as by those of the duck host (i.e., osteoclasts). This provides another neural crest‐dependent mechanism that contributes to the shorter beaks of quail and chimeric quck. In support of this conclusion, we find that when we experimentally apply small molecules, pharmacologic agents, or recombinant proteins to inhibit resorption we can lengthen the beak, whereas if we activate resorption we can shorten the beak.

By extension then, our work reveals that beak size in birds is inversely proportional to levels of bone resorption, and that such levels are established by neural crest mesenchyme. Prior work on Darwin's finches and other species, have correspondingly argued that the calcium binding protein *Calmodulin* is a key determinant of beak length (Abzhanov et al., 2006; Gunter, Koppermann, & Meyer, 2014; Schneider, 2007). Quite interestingly, *Calmodulin* is known to regulate osteoclasts and osteocytes (Choi, Ann, et al., 2013; Choi, Choi, Oh, & Lee, 2013; Seales, Micoli, & McDonald, 2006; Zayzafoon, 2006), calcium signaling is important for bone resorption (Hwang & Putney, 2011; Kajiya, 2012; Xia & Ferrier, 1996; Xiong et al., 2014), and this pathway can affect jaw size (Gunter et al., 2014; Parsons & Albertson, 2009). Thus, taken together, all of these studies imply that bone resorption may function like a rheostat during skeletal evolution, and in this capacity might be especially tuned to the availability of dietary calcium, to the effects of calcium‐dependent hormones, and to gradients of calcium signaling within the beak primordia (Schneider, 2007, 2018). Such spatial and temporal regulation of resorption by neural crest mesenchyme likely acts as a determinant of species‐specific size and shape by creating local zones of resorption in quail versus duck that more‐or‐less sculpt the bone and inhibit or promote directional growth.

Overall, we have discovered that neural crest mesenchyme wields precise spatial and temporal control over each step of osteogenesis including the induction, differentiation, deposition, mineralization, and resorption of bone (Ealba et al., 2015; Eames & Schneider, 2008; Hall et al., 2014; Merrill et al., 2008; Schneider & Helms, 2003). This control appears to be integrated and implemented on multiple interacting genetic and epigenetic levels (Alberch, 1982a) such that neural crest can orchestrate species‐specific programs for skeletal size and shape throughout development and serve as a source for morphological variation in the craniofacial complex during evolution. Most likely, the mechanisms that distinguish the species‐specific programs of quail from those of duck are multifactorial and based on intrinsic and emergent differences in genome organization, cis‐regulation of individual genes, epigenetic activities of non‐coding RNA at the transcriptional and post‐transcriptional level, connectivity at nodes within gene regulatory networks, biochemical interactions among gene products (e.g., enzymes and other proteins), post‐translational modification of proteins, diffusion‐reaction gradients and thresholds that affect induction and developmental potential, properties and movements of cells, and/or physical and signaling interactions among tissues (Schneider, 2018). Changes at any of these hierarchical levels of organization during development could undoubtedly be a means to affect species‐specific morphology. By investigating such changes in quail versus duck we aspire to rise to the challenge set forth by Alberch et al. (1979) when they expressed their hope that their “attempts to construct a quantitative theory will stimulate others to delve more deeply below the level of pure phenomenology and come to grips with the central issue underlying evolutionary diversification of size and shape—that is, the morphogenetic unfolding of genetic programs in ontogeny and their alteration in the course of phyletic evolution” (p. 297).

## 9 ORIGIN OF SPECIES‐SPECIFIC PATTERN IN THE JAW MUSCULATURE

In addition to cartilage and bone, cranial neural crest mesenchyme also produces skeletal and muscle connective tissues such as tendons, ligaments, fascia, and epi‐ and endomysia (Couly et al., 1993; Köntges & Lumsden, 1996; Le Lièvre & Le Douarin, 1975; Noden, 1978b, 1983; Noden & Schneider, 2006). Head and jaw muscles however, form from mesodermal mesenchyme (Couly et al., 1992; Evans & Noden, 2006; Noden, 1983; Noden & Francis‐West, 2006; Noden & Trainor, 2005; Scaal & Marcelle, 2018; Wachtler & Jacob, 1986). Given these differences in embryonic origin, the quail‐duck chimeric system provides a means to examine the extent to which donor neural crest mesenchyme regulates species‐specific pattern in host muscle (Fish & Schneider, 2014b; Solem et al., 2011; Tokita & Schneider, 2009). A broad range of prior investigations have revealed that cranial neural crest mesenchyme plays a critical role during muscle development. In particular, the early migration, differentiation, and spatial patterning of myogenic mesenchyme in the head relies on interactions with surrounding muscle connective tissues (Borue & Noden, 2004; Ericsson, Cerny, Falck, & Olsson, 2004; Francis‐West et al., 2003; Grammatopoulos et al., 2000; Grenier, Teillet, Grifone, Kelly, & Duprez, 2009; Hall, 1950; Knight, Mebus, & Roehl, 2008; Knight & Schilling, 2006; Köntges & Lumsden, 1996; McGurk et al., 2017; Nassari, Duprez, & Fournier‐Thibault, 2017; Noden, 1983, 1986a, 1988; Noden, Marcucio, Borycki, & Emerson, 1999; Noden & Schneider, 2006; Noden & Trainor, 2005; Olsson, Falck, Lopez, Cobb, & Hanken, 2001; Pasqualetti et al., 2000; Rinon et al., 2007; Schilling et al., 1996; Schnorrer & Dickson, 2004; Subramanian & Schilling, 2015; Sugii et al., 2017; Tokita, Nakayama, Schneider, & Agata, 2013; Trainor & Krumlauf, 2000; Trainor, Sobieszczuk, Wilkinson, & Krumlauf, 2002; Tzahor et al., 2003).

**Figure 3:**
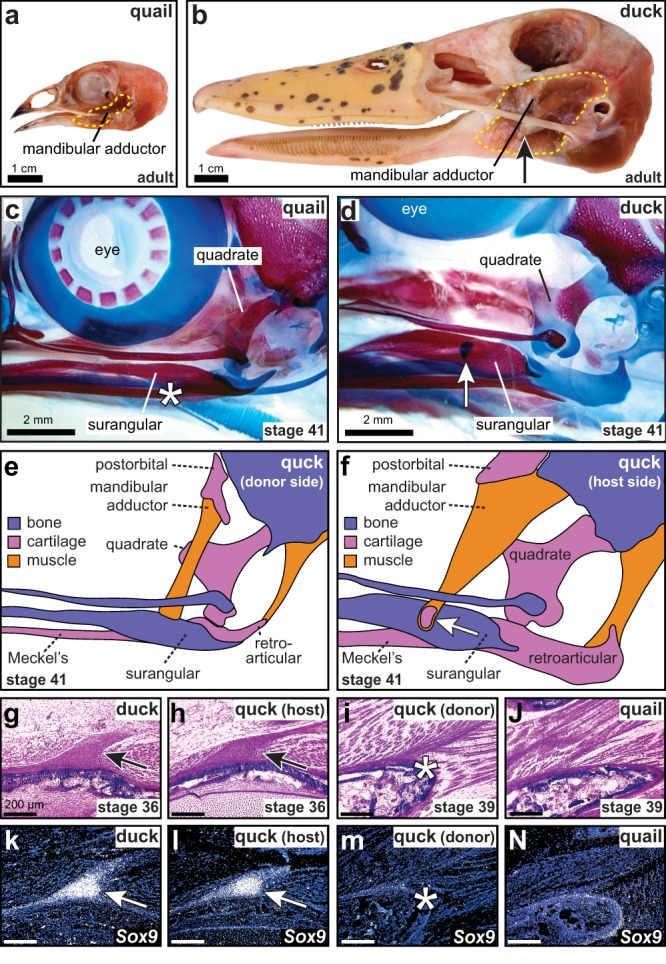
**(a)** Adult quail and **(b)** duck heads in lateral view showing the mandibular adductor muscles that close the jaw (yellow dashed lines). The duck mandibular adductor inserts more laterally and proximally, and integrates into a pronounced coronoid process along the side of the lower jaw (black arrow) whereas the quail mandibular adductor inserts more dorsally and distally. Modified from Tokita and Schneider (2009). **(c)** Lateral view of cleared and stained quail and **(d)** duck embryos at stage 41. Cartilage is blue and bone is red. In duck, the coronoid process forms as a secondary cartilage (white arrow) on the lateral side of the surangular bone along the lower jaw skeleton. A corresponding cartilage is absent in quail (white asterisk). **(e)** In quck chimeras, jaw muscles come from the duck host whereas skeletal and connective tissues come from quail donor neural crest. Jaw anatomy on the donor side is transformed to something more like that found in quail. The mandibular adductor is narrower, inserts dorsally along the surangular, and as in quail, does not contain secondary cartilage. **(f)** In contrast, on the host side of quck, the mandibular adductor muscle is broader and inserts laterally on the surangular bone, and secondary cartilage forms within the insertion (white arrow) like that normally observed in duck. **(g)** Trichrome‐stained section of duck in lateral view showing the mandibular adductor muscle at its insertion (black arrow and stained purple), which is wide and triangular shaped along the surangular bone (stained blue). **(h)** On the host side of quck, the insertion looks the same as in duck whereas **(i)** on the donor side of quck (white asterisk) and in **(j)** quail the insertion is relatively thin. **(k)** Coincident with the eventual formation of secondary cartilage in duck but not quail, the chondrogenic transcription factor S*ox9* is expressed highly in the insertion (white arrow) of duck and **(l)** on the host side of quck, but not in the insertion **(m)** on the donor side of quck (white asterisk) or **(n)** in quail. Modified from Solem et al. (2011)

With regard to the species‐specific patterning of the muscles, a clear example of the effects of neural crest mesenchyme can be seen in the resultant jaw complex of quail‐duck chimeras. Quail and duck have highly specialized jaw morphologies associated with their species‐specific modes of feeding. Quail use their sharp, pointed beaks like forceps to peck at seed on the ground whereas duck use a suction‐pump mechanism and apply leverage across their long, broad bills to strain water and sediment. Such differences in feeding behavior are mirrored in the size, shape, and attachment sites of their jaw muscles as well as in their jaw kinetics and mechanics (Bout & Zweers, 2001; Dawson, Metzger, Baier, & Brainerd, 2011; Fisher, 1955; Soni, 1979; Zweers, 1974; Zweers, Gerritsen, & Kranenburg‐Voogd, 1977; Zweers, Kunz, & Mos, 1977) (Figure 3a–d). In quail‐duck chimeras we find that quail donor neural crest mesenchyme imparts quail‐like pattern on the duck host mesoderm‐derived jaw muscles (Solem et al., 2011; Tokita & Schneider, 2009). These transformations are not only species‐specific but also stage‐specific, in that the muscle anatomy on the donor side is more like that found in control quail three stages later. For example, in duck, the mandibular adductor muscle inserts on the lateral side of the mandible (Zweers, 1974; Zweers, Kunz, et al., 1977), whereas in quail, the same muscle inserts on the dorsal surface of the mandible (Baumel, 1993; Van den Heuvel, 1992). In quck chimeras, these species‐specific differences in the shape, orientation, and insertion point of the mandibular adductor muscle are patterned by neural crest mesenchyme. In particular, we find that quail donor neural crest mesenchyme is distributed throughout the skeletal and muscular connective tissues that surround the developing host muscle precursors and causes the duck host jaw muscles to elongate rostrally and attach dorsally as in quail (Solem et al., 2011; Tokita & Schneider, 2009; Woronowicz et al., 2018) (Figure 3e–j).

Preceding these dramatic morphological transformations are changes to the spatial and temporal patterns of gene expression for muscle connective tissue markers such as *Tcf4*, which is a transcription factor that functions downstream of the WNT pathway, and which plays an essential role during muscle development (Anakwe et al., 2003; Bonafede, Kohler, Rodriguez‐Niedenfuhr, & Brand‐Saberi, 2006; Mathew et al., 2011; Miller et al., 2007) including ordaining the spatial pattern of limb muscles (Kardon, Harfe, & Tabin, 2003). Another transcription factor, *Scleraxis* (*Scx*), which is expressed by and required for the differentiation of tendon and ligament progenitors in the trunk and head (Berthet et al., 2013; Blitz, Sharir, Akiyama, & Zelzer, 2013; Blitz et al., 2009; Brent, Braun, & Tabin, 2005; Cserjesi et al., 1995; Grenier et al., 2009; Murchison et al., 2007; Pryce, Brent, Murchison, Tabin, & Schweitzer, 2007; Schweitzer et al., 2001; Shukunami, Takimoto, Oro, & Hiraki, 2006), is also up‐regulated on the donor side of quck chimeras in quail‐like patterns. Thus, these neural crest‐mediated changes in gene expression appear to direct the species‐specific shape and attachment sites of the jaw muscles. This in turn, alters the functional morphology and the associated mechanical force environment such that the duck host side of quck chimeras induces *Sox9* expression and a robust secondary cartilage at the insertion of the mandibular adductor muscle on the coronoid process of the mandible, whereas the quail donor side fails to express *Sox9* or form a secondary cartilage just like in control quail (Solem et al., 2011; Woronowicz et al., 2018) (Figure 3k–n).

Interestingly, donor neural crest mesenchyme does not seem to alter the early programs for host myogenic specification or muscle differentiation, which based on our expression analyses of early muscle markers and structural proteins continue to follow the timetable of the host (Tokita & Schneider, 2009). Such a result is not entirely surprising given that muscle is an ancient mesodermal lineage that evolved long before the appearance of the cranial neural crest and therefore, likely executes aspects of its own developmental program rather autonomously. This also appears to be the case for the mesodermally‐derived blood vessels of the host, which are similarly unaffected in chimeras (Hall et al., 2014). However, this situation is quite unlike what we have observed for the donor‐derived programs for cartilage, bone, and tendon, and the host‐derived programs for epidermis (i.e., feathers and egg teeth) and osteoclasts, which in chimeras all follow in lockstep with the quail donor timetable and become accelerated by three stages (Ealba et al., 2015; Eames & Schneider, 2005, Eames & Schneider, 2008; Hall et al., 2014; Merrill et al., 2008; Schneider & Helms, 2003).

In sum, neural‐crest mesenchyme and its muscle connective tissue derivatives transmit species‐specific patterning information to craniofacial muscles by executing autonomous molecular programs and by dominating interactions with their partners from the mesoderm. Such mechanistic insights help explain how skeletal and muscular components in the jaw complex have so intimately co‐evolved as species radiate into new niches and their mouthparts become adapted in various ways. This can be seen clearly in birds such as parrots, where the number and organization of jaw muscles have been extremely modified, and most likely in close association with changes to neural crest (Tokita, 2004, 2006; Tokita, Kiyoshi, & Armstrong, 2007; Tokita et al., 2013; Zusi, 1993). The fact that neural crest mesenchyme establishes a direct relationship between skeletal anatomy, muscle architecture, and feeding mechanics suggests that the capability of a given species to modify its jaw complex rapidly during evolution, which is critical to accommodate novel ecological conditions, resides in the cranial neural crest. Thus, the neural crest has played a leading role in dictating species‐specific pattern and in directing the structural and functional integration of the craniofacial complex during the course of vertebrate evolution.

## 10 CONCLUSION

The vertebrate craniofacial complex displays tremendous conservation in its anatomical organization but remarkable diversity in its species‐specific size and shape. Such dualism mirrors the need for the craniofacial complex to satisfy essential requirements for survival like feeding, breathing, and sensing, yet simultaneously generate enough morphological variation to allow for adaptive evolution. Conspicuously, much of the evolutionary diversity in the craniofacial complex has appeared in those structures derived from the cranial neural crest, suggesting a high degree of plasticity (Eames & Schneider, 2005; Fish & Schneider, 2014b; Gans & Northcutt, 1983; Hanken & Gross, 2005; Jheon & Schneider, 2009; Le Douarin et al., 2004; Noden & Schneider, 2006; Northcutt, 2005; Schlosser & Wagner, 2004; Schneider, 1999, 2005, 2015; Trainor et al., 2003; West‐Eberhard, 1989, 2003; Young et al., 2014).

To this point, one might ask, what endows the cranial neural crest with both the plasticity to drive evolutionary diversification and the regulatory abilities to put them near the top of hierarchies in developmental programs for species‐specific pattern? Contributing factors may include gene duplication events, which are believed to have capacitated the evolution of neural crest development via co‐option and novel gene function (Green & Bronner, 2013; Meulemans & Bronner‐Fraser, 2005). Similarly, results from transcriptional profiling studies point to novel signaling pathways and suites of transcription factors that are enriched in sub‐populations of neural crest (Lumb, Buckberry, Secker, Lawrence, & Schwarz, 2017; Simoes‐Costa & Bronner, 2016; Simoes‐Costa, Tan‐Cabugao, Antoshechkin, Sauka‐Spengler, & Bronner, 2014). Moreover, given that cranial neural crest derivatives participate deeply in the development and patterning of multiple systems including the nervous, neuroendocrine, integumentary, and skeletal, regulatory changes to the neural crest can be a major source of simultaneous evolutionary transformations in behavior, pigmentation, as well as the size and shape of cartilage and bone in the face (Lord et al., 2016; Sanchez‐Villagra et al., 2016; Schneider, 2005, 2018; Singh et al., 2017; Wilkins et al., 2014).

Without a doubt, data from a wide variety of experimental systems and strategies performed in diverse vertebrate taxa, demonstrate that neural crest mesenchyme controls species‐specific pattern. Work from our lab takes this conclusion one step further and reveals that neural crest does so by autonomously executing molecular and cellular programs for making neural crest‐derived structures, as well as by governing the required interactions with adjacent tissues (e.g., ectodermal epithelia and mesodermal mesenchyme). Furthermore, the precise origin of species‐specific pattern is rooted in the fact that epithelia are generally permissive and supply generic anatomical information (e.g., “form a lower jaw”) while neural crest mesenchyme contains instructive information for size and shape (e.g., “form a quail‐like lower jaw” versus “form a duck‐like lower jaw”). In other words, the list of parts is more or less the same but the species‐specific differences that arise during the construction process appear to stem from where, when, and for how long neural crest mesenchyme autonomously activates and executes intrinsic molecular programs including the expression of receptors that allow for signal transduction to begin and end, as well as a variety of transcription factors that modulate gene regulatory networks. Ultimately, the developmental fate of each species is determined by the differential unfurling of each genome in three dimensions as a function of absolute and/or relative time (e.g., cell cycle dynamics and maturation rates).

Most significantly, the propensity of neural crest mesenchyme to keep track concurrently of stage‐specific and species‐specific time, size, and shape provides a potent mechanism linking ontogeny and phylogeny in the integumentary and musculoskeletal systems, both of which have been critical to the success of vertebrates. Such findings about the integration of these systems vis‐à‐vis the cranial neural crest also reveal the many ways development can play a “generative and regulatory” role in the evolution of species‐specific pattern (Alberch, 1982b), and they help substantiate heterochrony as a viable developmental mechanism whereby species‐specific transformations in size and shape can come about via changes in the timing of developmental events (Alberch et al., 1979; De Beer, 1930; Fish & Schneider, 2014b; Hall, 1984; Schneider, 2018; Smith, 2003).

In this framework, a major remaining question for future research involves identifying on a much larger scale (i.e., systems level) where, when, and how variation in the molecular and cellular programs that are directed by the neural crest leads to species‐specific changes in size and shape. Addressing this question has important implications for understanding both evolution and the etiologies of craniofacial birth defects (Schneider, 2015). Furthermore, heterochrony may be an oversimplification of the many processes at work and instead a more multidimensional strategy that accounts for the effects of complex changes in facets such as the levels and spatial distribution of gene expression over developmental time (Depew & Simpson, 2006) may be necessary. Additional approaches that incorporate genome‐wide differences in the regulation of neural crest‐mediated programs among divergent taxa, have great potential to elucidate these issues (Betancur, Bronner‐Fraser, & Sauka‐Spengler, 2010; Long, Prescott, & Wysocka, 2016; Nikitina et al., 2008; Prescott et al., 2015; Rebeiz & Tsiantis, 2017; Sauka‐Spengler & Bronner‐Fraser, 2008; Sauka‐Spengler et al., 2007; Trinh et al., 2017; Williams et al., 2018). One can only imagine what the next 150 years of neural crest biology will uncover.
